# Utilizing a nomogram to predict the one-year postoperative mortality risk for geriatric patients with a hip fracture

**DOI:** 10.1038/s41598-023-38297-1

**Published:** 2023-07-08

**Authors:** Cheng-Yi Wu, Ching-Fang Tsai, Hsin-Yi Yang

**Affiliations:** 1 Department of Orthopedics, Ditmanson Medical Foundation Chia-Yi Christian Hospital, Chiayi City, Taiwan; 2 Osteoporosis Center, Ditmanson Medical Foundation Chia-Yi Christian Hospital, Chiayi City, Taiwan; 3 Department of Medical Research, Clinical Data Center, Ditmanson Medical Foundation Chia-Yi Christian Hospital, No. 539, Zhongxiao Rd., East District, Chiayi City, 60002 Taiwan

**Keywords:** Diseases, Medical research, Risk factors

## Abstract

Despite the abundance of research on the risk factors for mortality following hip fracture surgery, there has been a dearth of studies on prediction models in this population. The objective of this research was to explore the influencing factors and construct a clinical nomogram to predict one-year postoperative mortality in patients with hip fracture surgeries. Using the Ditmanson Research Database (DRD), we included 2333 subjects, aged ≥ 50 years who underwent hip fracture surgery between October, 2008 and August, 2021. The endpoint was all-cause mortality. A least absolute shrinkage and selection operator (LASSO) derived Cox regression was performed to select the independent predictors of one-year postoperative mortality. A nomogram was built for predicting one-year postoperative mortality. The prognostic performance of nomogram was evaluated. On the basis of tertiary points in a nomogram, the patients were divided into low, middle and high risk groups, and compared by the Kaplan–Meier analysis. Within 1 year after hip fracture surgery, 274 patients (11.74%) died. Variables retained in the final model comprised age, sex, length of stay, RBC transfusions, hemoglobin, platelet, and eGFR. The AUC for one-year mortality predictions were 0.717 (95% CI = 0.685–0.749). The Kaplan–Meier curves were significantly different among the three risk groups (p < 0.001). The nomogram showed good calibration. In summary, we explored the one-year postoperative mortality risk in geriatric patients with a hip fracture and developed a prediction model that could help clinicians identify patients at high risk of postoperative mortality.

## Introduction

Hip fracture is a major and increasing global health concern. Based on the epidemiologic projections, it is estimated that there will be 2.6 million individuals will suffer a hip fracture by 2025^1^. The number of hip fractures expected to increase to 6.3 million by 2050 as life expectancy of the world population increases^2^. They have been associated with increased morbidity, loss of independence, high rate of institutionalization, and mortality^3^. Mortality is considered the most serious consequence, the one-year mortality after hip fractures surgery is high, ranging between 15 and 36%^4–7^. Therefore, it is critical to explore prognostic factors to identify patients with hip fracture who have a high risk of death as early as possible.

Advanced age, male sex, high comorbidity burden, frailty, low functional status or reduced mobility, cognitive impairment and time-to-surgery have been identified as significant predictors of postoperative mortality in hip fracture patients^3,5,8^. In spite of this knowledge, it could be a challenge to obtain an overview risk profile of each patient in clinical practice. In view of the importance of early identification of adverse prognosis in hip fracture for patient treatment and management, an increasing number of researchers and policymakers tend to rely on predictive models.

As far as we know, several research groups have developed surgery-specific or hip fracture-specific risk prediction models^9,10^. For instance, the Orthopaedic version of the Physiologic and Operative Severity Score for the enUmeration of Mortality and morbidity (O-POSSUM)^11^ and The Estimation of Physiologic Ability and Surgical Stress (E-PASS)^12,13^ can be aid to predict mortality, however, their intricate formulas are not easy to apply in a busy clinical setting and some of these data in these models are not easily available and must be specifically collected^14^. Some models which are specifically designed for early mortality after hip fracture [as the Nottingham Hip Fracture Score (NHFS)]^13,15^ are simple and faster to use, however none of them showed excellent discrimination.

While predictors for postoperative mortality of hip fracture have been diffusely studied, research pertaining to risk prediction models is limited. The nomogram has been accepted as a reliable tool to create a simple intuitive graph of a statistical predictive model that quantifies the risk of a clinical event. Herein, we conducted this retrospective study to investigate the predictors for one-year postoperative mortality and constructed a corresponding clinical nomogram to predict high-risk ones.

## Patients selection and methods

### Data source

We conducted a retrospective cohort study of hip fracture surgery patients with the data retrieved from the electronic medical record database [Ditmanson Research Database (DRD)] at Ditmanson Medical Foundation Chia-Yi Christian Hospital, a regional hospital in southern Taiwan.

### Ethics statement

This study was approved by the Institutional Review Board of the Ditmanson Medical Foundation Chia-Yi Christian Hospital, Taiwan (CYCH-IRB No: 2019063). Informed consent was waived by the Institutional Review Board of the Ditmanson Medical Foundation Chia-Yi Christian Hospital because of the retrospective nature of the study and the analysis used anonymous clinical data. All of the methods were performed in accordance with relevant guidelines and regulations, including any relevant details.

### Patients selection

A total of 2333 patients underwent hip fracture surgery between October, 2008 and August, 2021. The eligibility criteria were: (1) ages ≥ 50 years; (2) introchanteric (ICD-9-CM Code 820.2, 820.20, 820.21, 820.3, 820.30, 820.31; ICD-10 Code S72.1), subtrochanteric (ICD-9-CM Code 820.22, 820.32; ICD-10 Code S72.2), or femoral neck fractures (ICD-9-CM Code 820.0, 820.1, 820.8, 820.9; ICD-10 Code S72.0); (3) recipients of surgeries including partial hip replacement, open or closed reduction internal fixation; (4) caused by a low-energy injury (such as osteoporosis and fall). We excluded patients who were below age 50 years, patients with pathological fractures, multiple fractures or multiple trauma, conservative treatment, and loss of data.

### Predictor variables and primary outcome

The following patient demographics were extracted from the DRD: age, sex, length of stay, intensive care unit (ICU), operation time, type of anaesthesia, RBC transfusions, surgical procedure performed, type of hip fracture, time from injury to operation, preoperative clinical variables including comorbidities [eg. diabetes mellitus (DM), hypertension, congestive heart failure, chronic obstructive pulmonary disease (COPD), chronic kidney disease (CKD), cancer, and dementia], and admission laboratory data (eg. RBC, WBC count, hemoglobin, hematocrite, platelet, BUN, creatinine, and eGFR). The estimated glomerular filtration rate (eGFR) was determined according to serum Cr levels and sex using the calculation method used in a previous study^16^. The main outcome was the occurrence of death at one-year after surgery for hip fracture. The dates of mortality were obtained in reference to the Taiwan Death Registry database.

### Statistical analysis

All statistical analyses were performed using the R programming language and environment (http://www.r-project.org/). Results were considered statistically significant for a 2-tailed test if p-value < 0.05. Continuous variables were presented as the mean ± standard deviation (SD), and were compared using the Student's t test whereas categorical variables was presented by percentages and comparisons of categorical data were examined by Chi square test. If a potential predictor had more than 5% missing data, multiple imputation was used as a method to handle the missing values^17^. We divided continuous variables into classification variables, which makes the model more objective and simpler. The cut-off value of the classification variable was age, length of stay, operation time and the normal value of clinical laboratory examination. The cut-off value of numeric values as follows: age was 85 years, length of stay was 15 days, operation time was 90 min, WBC was 3.5 and 9.9*10^3^/μL, hemoglobin was 10 g/dL, RBC transfusions was 2 units, platelet was 100*10^9^/L and eGFR was 60 mL/min/1.73 m^2^. The duration of hospitalization was assessed in terms of days, and was determined by calculating the difference between the date of admission and the date of discharge^18^. Patients who had a hospital stay of 15 days or more were classified as having a prolonged length of stay^19–21^. A least absolute shrinkage and selection operator (LASSO) derived Cox regression followed by ten-fold cross-validation of variables were used to identify the predictors for one-year postoperative mortality. Multivariate Cox regression was further performed to assess the prognostic value of selected variables. Univariate and multivariate Cox proportional hazards models were used to calculate hazard ratios (HRs) and 95% confidence interval (CI) for one-year postoperative mortality. Survival curves were depicted using the Kaplan–Meier method. These variables were further applied to build a nomogram for estimating the one-year survival ratios of one-year postoperative mortality using the rms package in R. The concordance index (C-index) and AUC curve were used to appraise the performance of the nomogram. After internal validation by bootstrapping, a calibration curve was used to evaluate the calibration of the nomogram by contrasting the actual risk and predicted risk. According to the net benefit and threshold probabilities, the clinical usefulness of the nomogram was estimated using the decision curve analysis (DCA).

## Results

### Patients’ characteristics

The demographics and clinical characteristics in the hip fracture patients who were alive and dead one-year postoperatively are compared in Table 1. Patients who died within one-year postoperatively tended to be older (81.09 years vs 77.73 years, p < 0.001), more often male (47.45% vs 30.45%, p < 0.001), longer length of stay (8.72 days vs 6.51 days, p < 0.001). Trochanteric fractures, spinal anaesthesia alone, RBC transfusions > 2 units and time from injury to operation ≧ 48 h were slightly more common among patients who died (p < 0.05), while the operation type and operation blood loss did not differ significantly (p > 0.05). There were significant difference in the comorbidities, including congestive heart failure, chronic kidney disease, and cancer, and in laboratory parameters, including RBC hemoglobin, hematocrite, BUN, creatinine and eGFR (p < 0.05) between survivors and nonsurvivors.

**Table 1:** Demographics and clinical characteristics of hip fracture patients who were alive and dead one-year postoperatively.

	Survivors	Nonsurvivors	p-value
Number	2059	274	
Age	77.73 ± 9.21	81.09 ± 9.38	< 0.001
< 85	1568 (76.15)	171 (62.41)	
≧ 85	491 (23.85)	103 (37.59)	
Sex			< 0.001
Female	1432 (69.55)	144 (52.55)	
Male	627 (30.45)	130 (47.45)	
Length of stay	6.51 ± 3.82	8.72 ± 7.57	< 0.001
≦ 15	2012 (97.72)	246 (89.78)	
> 15	47 (2.28)	28 (10.22)	
ICU			< 0.001
No	2005 (97.38)	252 (91.97)	
Yes	54 (2.62)	22 (8.03)	
Operation time (mins)			0.120
< 90	797 (38.71)	120 (43.80)	
≧ 90	1262 (61.29)	154 (56.20)	
Type of anaesthesia			0.003
General anaesthesia	1475 (71.64)	172 (62.77)	
Spinal anaesthesia alone	584 (28.36)	102 (37.23)	
Operation blood loss (mL)	88.73 ± 152.17	74.37 ± 166.05	0.147
RBC transfusions, unit			< 0.001
≦ 2	1537 (74.65)	161 (58.76)	
> 2	522 (25.35)	113 (41.24)	
Operation type			0.167
Closed reduction of fracture with internal fixation, femur	881 (42.79)	130 (47.45)	
Open reduction of fracture with internal fixation, femur	389 (18.89)	55 (20.07)	
Partial hip replacement	789 (38.32)	89 (32.48)	
Type of hip fracture			0.006
Femoral neck	1075 (52.21)	117 (42.70)	
Trochanteric	799 (38.81)	134 (48.91)	
Subtrochanteric	125 (6.07)	12 (4.38)	
Muti-sites	60 (2.91)	11 (4.01)	
Time from injury to operation ≧ 48 h			< 0.001
No	1860 (90.34)	230 (83.94)	
Yes	199 (9.66)	44 (16.06)	
Comorbidity			
DM	301 (14.62)	51 (18.61)	0.100
Hypertension	506 (24.58)	75 (27.37)	0.352
Congestive heart failure	14 (0.68)	6 (2.19)	0.028
COPD	71 (3.45)	13 (4.74)	0.363
CKD	36 (1.75)	17 (6.20)	< 0.001
Cancer	42 (2.04)	14 (5.11)	0.004
Dementia	36 (1.75)	7 (2.55)	0.488
Laboratory parameters^a^			
RBC	4.06 ± 0.67	3.74 ± 0.70	< 0.001
Normal	958 (46.53)	76 (27.74)	
Abnormal	1101 (53.47)	198 (72.26)	
WBC count	9.92 ± 3.62	9.84 ± 4.05	0.731
≧ 3.5	2051 (99.61)	271 (98.91)	
< 3.5	8 (0.39)	3 (1.09)	
Hematocrite	36.00 ± 5.29	33.41 ± 5.63	< 0.001
Normal	858 (41.67)	56 (20.44)	
Abnormal	1201 (58.33)	218 (79.56)	
Hemoglobin	11.98 ± 1.88	11.11 ± 1.96	< 0.001
≧ 10	1764 (85.67)	198 (72.26)	
< 10	295 (14.33)	76 (27.74)	
Platelet	201.79 ± 72.21	196.03 ± 83.64	0.224
≧ 100	1989 (96.60)	252 (91.97)	
< 100	70 (3.40)	22 (8.03)	
BUN	22.43 ± 12.71	30.31 ± 19.07	< 0.001
Creatinine	1.17 ± 1.17	1.81 ± 1.77	< 0.001
eGFR	76.59 ± 37.56	60.86 ± 41.61	< 0.001
≧ 60	1356 (65.86)	120 (43.80)	
< 60	703 (34.14)	154 (56.20)	

ICU, intensive care unit; RBC, red blood cells; DM, diabetes mellitus; COPD, chronic obstructive pulmonary disease; CKD, chronic kidney disease; WBC, white blood cells; Bun, blood urea nitrogen; eGFR, estimated glomerular filtration rate.

^a^Preoperative testing.

### Predictive factor selection

**Figure 1:**
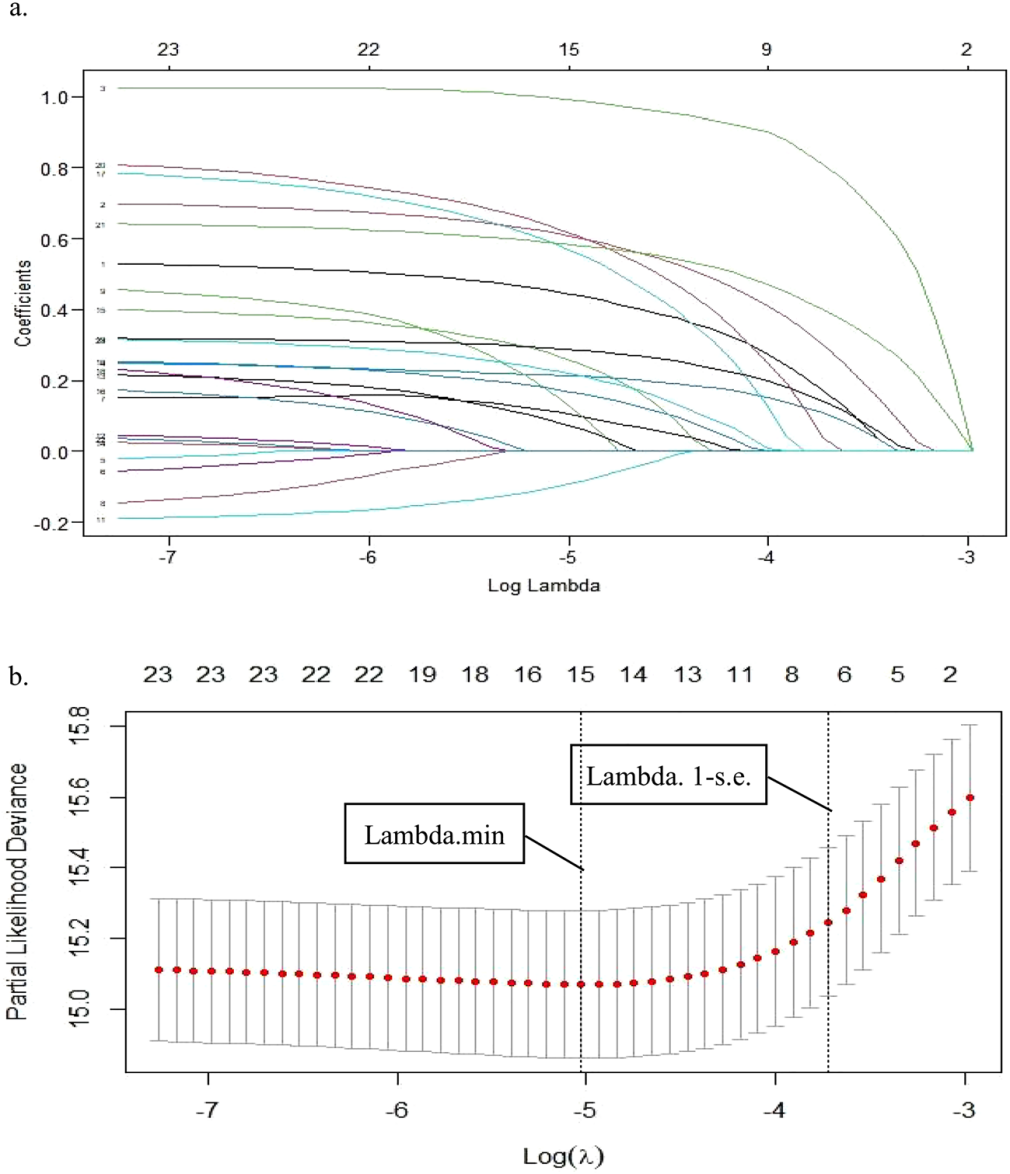
(**a**) Predictor selection using the LASSO Cox regression analysis; (**b**) seven risk factors selected using LASSO Cox regression analysis. The two dotted vertical lines were drawn at the optimal scores by minimum criteria and 1-s.e. criteria (at minimum criteria including age, sex, length of stay, RBC transfusions, hip fracture type, type of anaesthesia, operation time, diabetes mellitus, congestive heart failure, chronic obstructive pulmonary disease, cancer, dementia, hemoglobin, platelet, eGFR and WBC count; at 1-s.e. criteria including age, sex, length of stay, RBC transfusions, hemoglobin, platelet, and eGFR).

LASSO regression identified 7 predictors associated with postoperative mortality after hip fracture from 19 demographics and clinical characteristics, including age, sex, length of stay, RBC transfusions, hemoglobin, platelet and eGFR (Fig. 1). Then, we presented these predictors into multiple cox proportional hazard regression to quantify the association strength with postoperative mortality after hip fracture (Table 2).

**Table 2:** Univariate and multivariate cox hazard analysis of predictors for one-year postoperative mortality.

		Crude HR (95% CI)	p-value	Adjusted HR (95% CI)	p-value
Age	< 85	1.00		1.00	
	≧ 85	1.85 (1.45–2.36)	< 0.001	1.79 (1.39–2.30)	< 0.001
Sex	Female	1.00		1.00	
	Male	1.97 (1.55–2.50)	< 0.001	2.13 (1.67–2.71)	< 0.001
Length of stay	≦ 15	1.00		1.00	
	> 15	4.03 (2.76–5.89)	< 0.001	3.17 (2.15–4.69)	< 0.001
RBC transfusions (unit)	≦ 2	1.00		1.00	
	> 2	1.98 (1.56–2.52)	< 0.001	1.33 (1.02–1.75)	0.037
Hemoglobin (g/dL)	≧ 10	1.00		1.00	
	< 10	2.16 (1.66–2.81)	< 0.001	1.43 (1.07–1.92)	0.017
Platelet (1000/μL)	≧ 100	1.00		1.00	
	< 100	2.28 (1.47–3.52)	< 0.001	2.04 (1.31–3.16)	0.002
eGFR (mL/min /1.73 m^2^)	≧ 60			1.00	
	< 60	2.35 (1.85–2.98)	< 0.001	2.02 (1.58–2.58)	< 0.001

RBC, red blood cells; eGFR, estimated glomerular filtration rate.

### Nomogram as a tool for visualization

**Figure 2:**
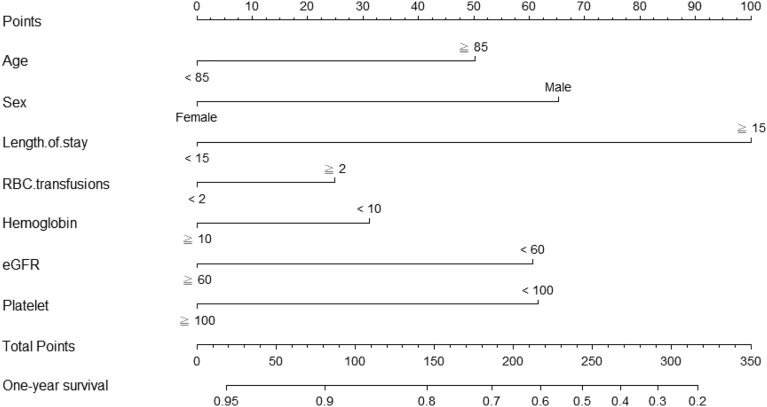
A nomogram for prediction of one-year mortality after surgery in patients with hip fractures.

To facilitate the clinical service, we converted the complex mathematical model into a nomogram (Fig. 2). It was necessary to sum the scores of variables included in the model. And then a vertical line at the total score was drawn and making it intersect with the one line representing the predicted mortality. The corresponding values of the point of intersection were the predicted one-year mortality of individuals. For example, a more than 85-year-old male patient, length of stay more than 15 days, RBC transfusions more than 2 unit, hemoglobin more than 10 g/dL, eGFR more than 60 mL/min /1.73 m^2^, and platelet more than 100 (1000/μL) had about a total score of 240, with a 1-year survival of 62% (Supplementary Table 1). It could be seen that nomogram was more convenient to use in clinical practice than mathematical formulas.

### Establishment of nomograms for predicting postoperative mortality

**Figure 3:**
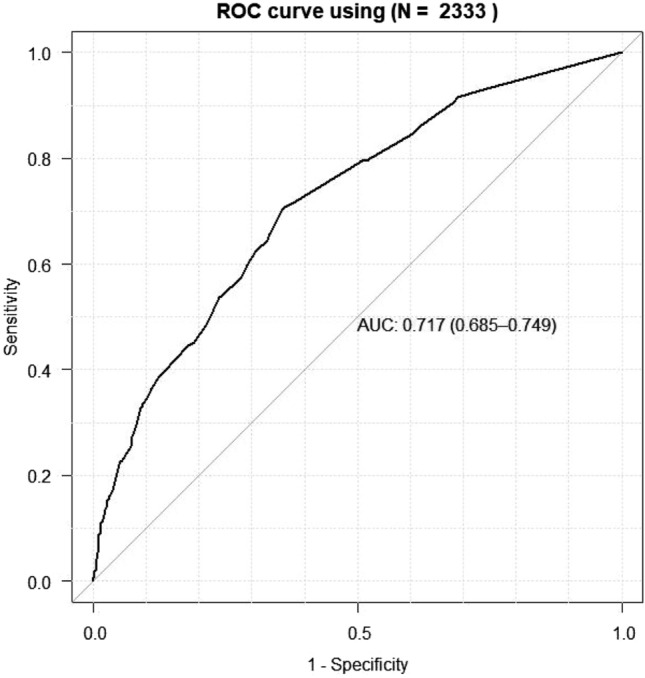
ROC curve for the one-year mortality prediction.

**Figure 4:**
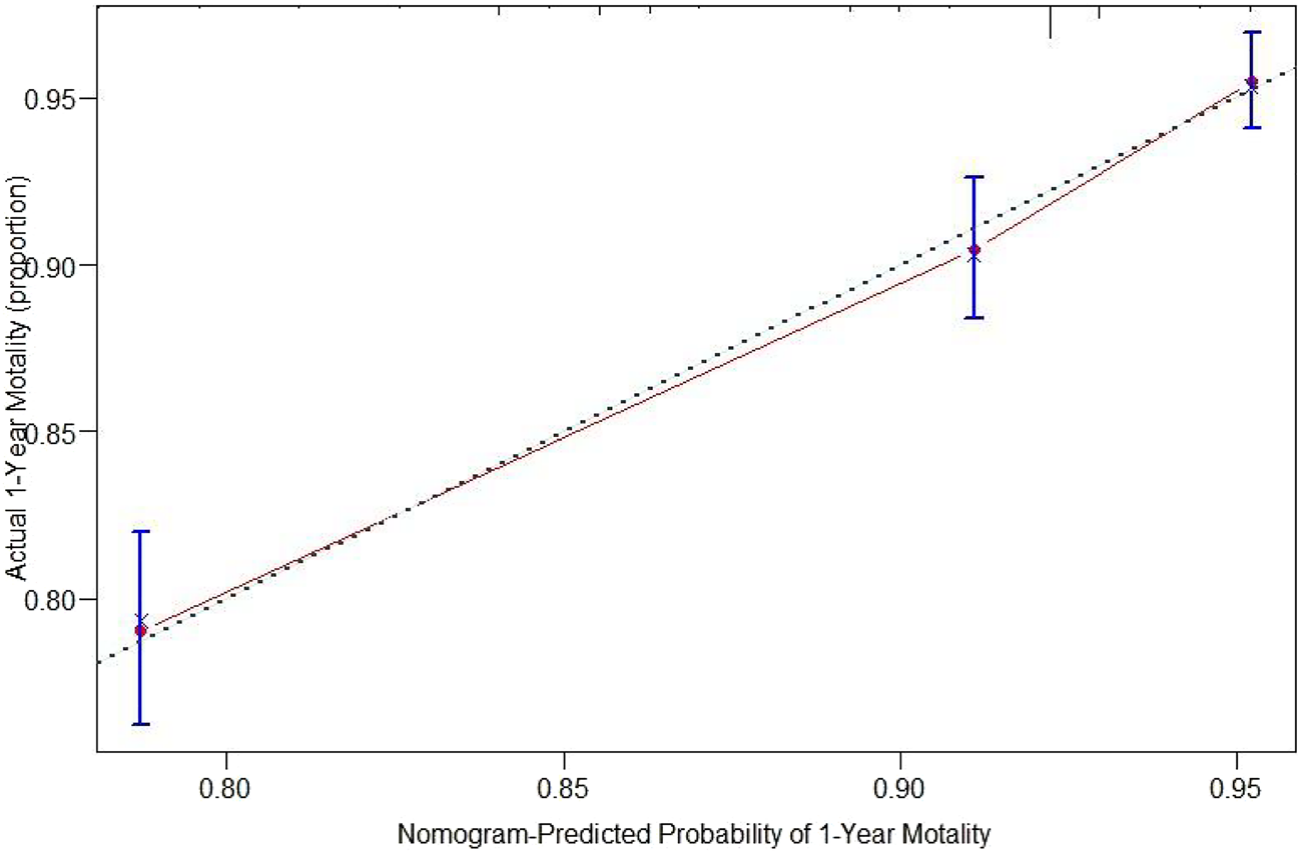
The calibration curves of the nomogram for predicting the overall survival rate after hip fracture surgery at 1-year.

**Figure 5:**
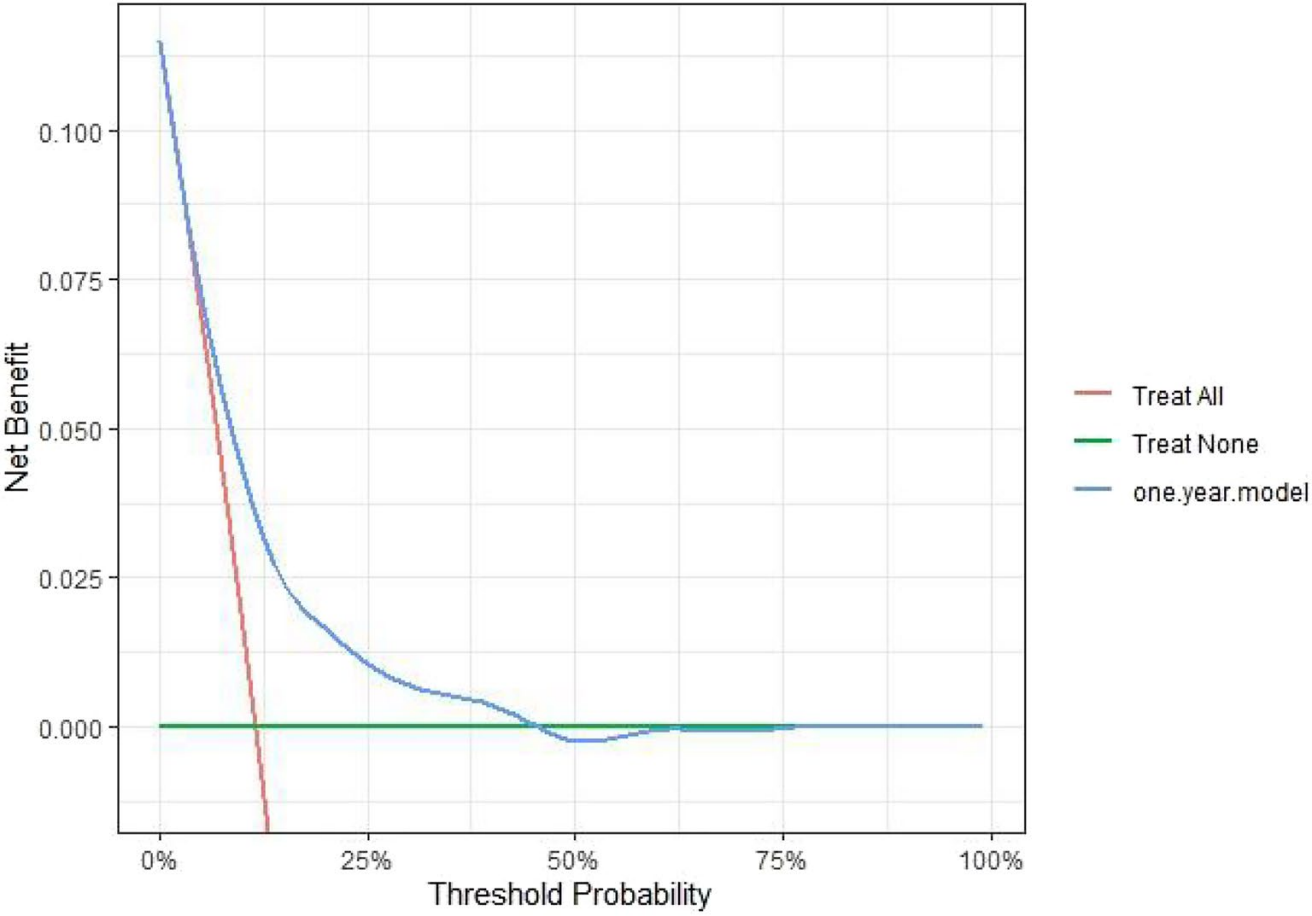
Decision curve analysis (DCA) curves.

The nomogram for predicting one-year survival was constructed based on the abovementioned predictors, as presented in Fig. 2. The predictive value of the nomogram was assessed by AUC. The AUC for one-year mortality predictions were 0.717 (95% CI = 0.685–0.749, Fig. 3). The calibration curves revealed good consistency between the nomograms’ predicted values and actual observations, with all calibration curve close to the 45-degree line (Fig. 4). DCA revealed that the nomogram had an excellent positive net clinical benefit within a specific threshold range, demonstrating the good clinical utility of nomogram (Fig. 5).

### Risk stratification based on the novel nomogram

**Figure 6:**
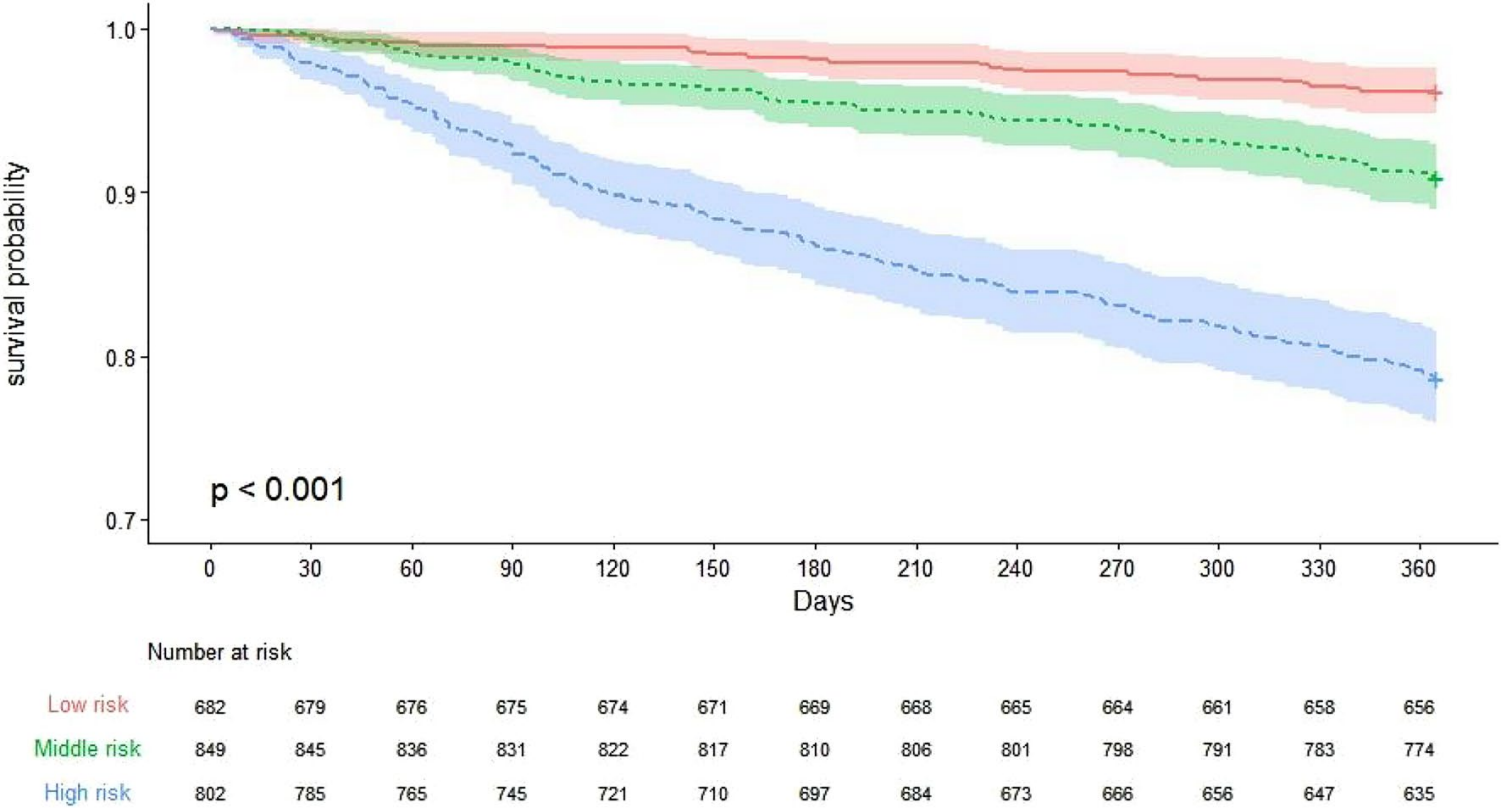
Kaplan–Meier survival curve of nomogram.

To reveal the independent discrimination ability of the simple-to-use postoperative mortality nomogram, we subdivided the patients into high- (score > 92), middle- (50—92), and low-risk (score < 50) groups according to the total risk scores in the study group. The patients in the three different risk subgroups showed significant differences in postoperatively mortality (p < 0.05, Fig. 6).

## Discussion

This retrospective cohort analysis used LASSO Cox regression analysis to identify independent risk factors of one-year postoperative mortality in geriatric patients with a hip fracture. The nomogram has been accepted as a reliable tool to create a simple intuitive graph of a statistical predictive model that quantifies the risk of a clinical event^22,23^. Numerous studies have indicated their potential value in clinical practice^24,25^. The following seven variables were included in the simplified model: age, sex, length of stay, RBC transfusions, hemoglobin, platelet and eGFR. This nomogram has a good discriminative and calibration capabilities. Moreover, all variables in the model are easily available, which guarantees the clinical utility of this nomogram.

As predicted, age and sex had an influence on mortality in patients with hip fracture. This study, as well as others, shows that mortality after hip fracture were increased with increasing age^19,26–28^. Moreover, multiple studies have shown the gender differences in mortality in patients with hip fracture. Our results are similar to other studies, that men had higher mortality risk than women. Reasons for sex differences remain unclear. Many scholars consider that male gender have poor lifestyle, like smoking and drinking, which are often associated with hypertension, coronary heart disease, COPD and other basic diseases, and their average life expectancy is shorter than that of female gender^29^. Therefore, males should be had higher mortality rates than females. Some have postulated that men seem to be sicker and frailer than women at this point in time of fracture, making them more vulnerable to postoperative mortality secondary to infections such as pneumonia and influenza^29,30^. Even though age and gender are non-modifiable factors, abundant comprehensive geriatric assessment, or intervention in geriatric male patients with hip fracture may be indispensable to diminish adverse outcomes.

The present study found that hemoglobin, platelet, and eGFR, which are easily accessible and convenient laboratory tests, reflected well the risk of mortality of patients after hip fracture surgery. The admission hemoglobin levels < 10 g/dL was found as a significant predictor of mortality in previous studies^31–33^. Our findings are concordance with those studies. These low hemoglobin concentration patients commonly also receives packed red cell blood transfusions during their hospital admission. Previous authors considered that the degree of anemia is a marker of underlying comorbid illness burden and physiologic reverse, so it perchance a significant mortality predictor^34^. The relationship between platelet and hip fracture remains unknown. Only one research has focus on preoperative platelet thresholds in orthopaedic trauma surgery and they found that a notably increased mortality at platelet levels below 150,000 μL, with the largest risk of complications observed at platelet counts < 100,000 μL^35^. Our results also showed that hip fracture with platelet levels below 100,000 μL had a higher risk of mortality than those ≧ 100,000 μL.

Previous studies also have presented that poor renal function is a predictor of mortality in hip fracture patients^36–39^. A study by Gulin et al.^40^ found that patients less than 85 years of age with eGFR < 55.4 mL/min/1.73 m^2^ had almost ten times higher mortality rates. A couple of studies denoted eGFR as a predictor of mortality in patients with hip fracture after surgery. According to a multivariate statistical analysis, presented an association between CKD stage 4 (CKD-EPI equation) and higher mortality risk at 1 year^41,42^. Our study did find that patients who had admission GFR < 60 mL/min/1.73 m^2^ had a mortality risk 2.01 times greater than those who had GFR more than 60 mL/min/1.73 m^2^. We considered hemoglobin concentration and GFR level to be modifiable factors. Because these possible causes of low hemoglobin concentration and low GFR level are potentially treatable and could reduce the mortality rate.

There are some limitations in our study. First, because of selection bias, our single-center retrospective study may limit the applicability of the model to other regions. Second, the nomogram showed medium prediction accuracy may suggest that other factors should be included. Some candidate variables were discarded because the missing values were greater than 20%. These may have inevitably caused bias. Finally, our predictive model lacks validation of an external population. Prospective, multicenter or population-based, large sample cohort studies are needed to validate our model further in the future.

### Conclusion and future directions

We constructed a convenient nomogram model to predict the postoperatively mortality of hip fracture patients at one-year, based on objective demographics and laboratory results, which can not only assist physicians with reasonable assessments and treatments but also help patients with consultation. The nomogram constructed suggests that age, sex, length of stay, RBC transfusions, hemoglobin, platelet, and eGFR are all significant predictors for one-year postoperative mortality risk in geriatric patients with a hip fracture.

## Supplementary Information

Supplementary Table 1.

## Data Availability

The data that support the findings of this study are available from Ditmanson Medical Foundation Chia-Yi Christian Hospital. Restrictions apply to the availability of these data, which were used under license for this study. Data are available from the corresponding author with the permission of Ditmanson Medical Foundation Chia-Yi Christian Hospital.

## Abbreviations

CI: Confidence interval
C-index: Concordance index
DCA: Decision curve analysis
DRD: Ditmanson Research Database
E-PASS: Estimation of Physiologic Ability and Surgical Stress
HRs: Hazard ratios
LASSO: Least absolute shrinkage and selection operator
NHFS: Nottingham Hip Fracture Score
O-POSSUM: Operative Severity Score for the enUmeration of Mortality and morbidity
SD: Standard deviation
