# Supplementary material for: Utilizing a nomogram to predict the one-year postoperative mortality risk for geriatric patients with a hip fracture
Source: Sci Rep. 2023 Jul 8;13:11091. doi: 10.1038/s41598-023-38297-1 (PMC10329653; doi:10.1038/s41598-023-38297-1)
Supplement: Supplementary file 1 — Supplementary Table 1. [file 41598_2023_38297_MOESM1_ESM.docx]

Supplementary Table 1. Predicted 1-year survival of a specific patient based on nomogram

|  | Variable value |  | Variable score |
| --- | --- | --- | --- |
| Age | ≧ 85 |  | 50 |
| Sex | Male |  | 65 |
| Length of stay | > 15 |  | 100 |
| RBC transfusions (unit) | > 2 |  | 25 |
| Hemoglobin (g/dL) | ≧ 10 |  | 0 |
| eGFR (mL/min /1.73m^2^) | ≧ 60 |  | 0 |
| Platelet (1,000/μL) | ≧ 100 |  | 0 |
| Total score |  | 240 |  |
| Predicted 1-year survival |  | 62% |  |

Abbreviation: RBC = Red blood cells; eGFR = estimated glomerular filtration rate
